# Tissue Damage in the Canine Normal Esophagus by Photoactivation with Talaporfin Sodium (Laserphyrin): A Preclinical Study

**DOI:** 10.1371/journal.pone.0038308

**Published:** 2012-06-13

**Authors:** Takahiro Horimatsu, Manabu Muto, Yusuke Yoda, Tomonori Yano, Yasumasa Ezoe, Shinichi Miyamoto, Tsutomu Chiba

**Affiliations:** Department of Gastroenterology and Hepatology, Graduate School of Medicine Kyoto University, Kyoto, Japan; National Taiwan University, Taiwan

## Abstract

**Background:**

Treatment failure at the primary site after chemoradiotherapy is a major problem in achieving a complete response. Photodynamic therapy (PDT) with porfimer sodium (Photofrin®) has some problems such as the requirement for shielding from light for several weeks and a high incidence of skin phototoxicity. PDT with talaporfin sodium (Laserphyrin) is less toxic and is expected to have a better effect compared with Photofrin PDT. However, Laserphyrin PDT is not approved for use in the esophagus. In this preclinical study, we investigated tissue damage of the canine normal esophagus caused by photoactivation with Laserphyrin.

**Methodology/Principal Findings:**

Diode laser irradiation was performed at 60 min after administration. An area 5 cm oral to the esophagogastric junction was irradiated at 25 J/cm^2^, 50 J/cm^2^, and 100 J/cm^2^ using a three-step escalation. The irradiated areas were evaluated endoscopically on postirradiation days 1 and 7, and were subjected to histological examination after autopsy. The areas injured by photoactivation were 52 mm^2^, 498 mm^2^, and 831 mm^2^ after irradiation at 25 J/cm^2^, 50 J/cm^2^, and 100 J/cm^2^, respectively. Tissue injury was observed in the muscle layer or even deeper at any irradiation level and became more severe as the irradiation dose increased. At 100 J/cm^2^ both inflammatory changes and necrosis were seen histologically in extra-adventitial tissue.

**Conclusions/Significance:**

To minimize injury of the normal esophagus by photoactivation with Laserphyrin, diode laser irradiation at 25 J/cm^2^ appears to be safe. For human application, it would be desirable to investigate the optimal laser dose starting from this level.

## Introduction

Photodynamic therapy (PDT) is a local endoscopic treatment using a photochemical reaction induced by an oncotropic photosensitizer and a laser [1]–[3]. PDT is useful for treating superficial esophageal cancer, preventing the development of adenocarcinoma from high-grade dysplasia in the Barrett’s esophagus, and alleviating stenosis caused by advanced esophageal cancer [4]–[6].

We have reported on the benefits of PDT as a salvage treatment for local residues and recurrence after definitive chemoradiotherapy (CRT) for esophageal cancer. Salvage PDT has excellent treatment outcomes for local residues and recurrence after CRT with a 59.5–62.0% complete response rate and a 5-year overall survival of 36.1% without severe adverse events [7], [8]. By contrast, salvage surgery after definitive CRT is associated with high postoperative mortality (>10%) [9], [10]. Accordingly, in carefully selected patients without metastasis, salvage PDT after definitive CRT is a potential curative treatment option that may improve quality of life and prolong survival.

PDT with porfimer sodium (Photofrin®), a hematoporphyrin derivative and the first clinically approved photosensitizer, has clinical disadvantages such as the requirement for shielding from light for 4–6 weeks and a high incidence (25%) of skin toxicity because of photosensitivity [11], [12]. PDT with mono-l-aspartyl chlorin e6 (NPe6, talaporfin sodium, Laserphyrin®), a second-generation photosensitizer, has advantages such as: 1) a shorter period of light shielding of about 2 weeks, 2) 10% incidence of skin toxicity because of reduced photosensitivity, and 3) expected treatment effect in the deep-lying tissue areas with a laser at 664 nm instead of at 630 nm as used with Photofrin PDT [13], [14].

The first two advantages are meaningful for patients because of the shorter period of shielding and lower incidence of toxicity, but the third is associated with opposite effects, i.e., an antitumor effect and an increased risk of an adverse event such as perforation, especially in the esophagus.

Applying Laserphyrin PDT as a salvage treatment of the deep-lying tissue seems to be an advantage for eliminating residual tumors. However, Laserphyrin PDT is approved only in Japan and only for superficial lung cancer. No investigation has reported on the safety of Laserphyrin PDT in esophageal cancer and the normal esophagus. Investigating the antitumor effects of photoactivation is also difficult because of the difficulty in developing esophageal cancer in large animal models. However, before we can apply Laserphyrin PDT to human esophageal disease, we must know whether it is safe as a treatment in the esophageal wall.

To investigate the relationship between the irradiation dose and tissue injury in the normal esophagus, we conducted a preclinical study of Laserphyrin PDT using a canine model.

## Results

### General Conditions

In the 25 J/cm^2^ irradiation group, one dog vomited 50 ml fluid, and all dogs in the 50 J/cm^2^ and 100 J/cm^2^ irradiation groups vomited; average volumes were 33 ml and 113 ml, respectively. There was a tendency for vomiting to become more severe as the irradiation dose increased.

Appetite loss was not observed in any dog in the 25 J/cm^2^ irradiation group but was apparent in all dogs in the 50 J/cm^2^ and 100 J/cm^2^ irradiation groups. A loss of body weight was observed on day 7 and was related to the irradiation dose: 8.3% in the 25 J/cm^2^ group, 11.2% in the 50 J/cm^2^ group, and 14.8% in the 100 J/cm^2^ group.

### Laboratory Data (Figure 1 and Table 1)

In the 25 J/cm^2^ group, the white blood cell (WBC) counts were 13.1, 12.3, and 12.7×10^3^/mm^3^ before irradiation and on days 1 and 7 after irradiation, respectively; these values did not differ significantly. In the 50 J/cm^2^ group, the WBC count increased from 10.7 to 17.9 on day 1 but returned to 12.4×10^3^/mm^3^ on day 7; the value on day 7 did not differ from that before irradiation. In the 100 J/cm^2^ group, the WBC count increased from 12.4 to 25.9×10^3^/mm^3^ on day 1 and remained at 23.1×10^3^/mm^3^ on day 7.

**Figure 1:**
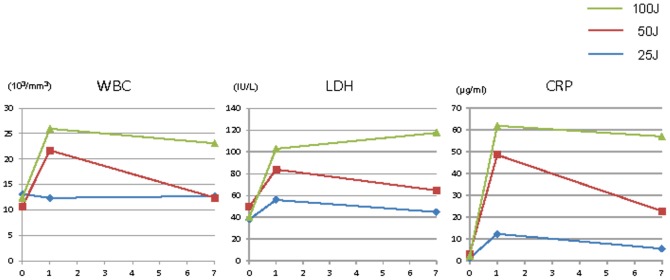
Laboratory data before and after irradiation. Compared with the baseline values, WBC count and CRP and LDH concentrations were increased with increasing radiation dose. WBC count and CRP and LDH concentrations returned to the baseline values on day 7 in the 25 J/cm^2^ group but remained high, especially in the 100 J/cm^2^ group.

**Table 1:** The injured areas and serum concentration of Laserphyrin.

Irradiation energy (J/cm^2^)	Animal No.	Injured area	Laserphyrin conc.
		(mean±SD, mm2)	(mean±SD, mm2)
25	1	52.3±47.9	24.0±0.67
	2		
	3		
50	4	498.3±430.7	24.6±22.18
	5		
	6		
100	7	831.0±691.7	23.0±9.37
	8		
	9		

The areas of tissue injury were 52.3±47.9 mm^2^, 498.3±430.7 mm^2^, and 831.0±691.7 mm^2^ after irradiation at 25 J/cm^2^, 50 J/cm^2^, and 100 J/cm^2^, respectively. These areas tended to be more extensive as the laser dose increased. The Laserphyrin concentration before irradiation ranged from 20 to 30 µg/ml in all groups.

In the 25 J/cm^2^ group, C-reactive protein (CRP) concentration increased from 1.2 to 12.3 mg/dl on day 1 and decreased to 5.5 mg/dl on day 7. In the 50 J/cm^2^ group, it increased from 3.2 to 48.6 on day 1 and decreased to 22.7 on day 7. In the 100 J/cm^2^ group, C-reactive protein concentration increased from 2.2 to 61.8 mg/dl on day 1 and remained high at 56.9 mg/dl on day 7.

In the 25 J/cm^2^ group, serum lactate dehydrogenase (LDH) level increased from 38.3 to 56.0 IU/L on day 1 and decreased to 45 IU/L on day 7. In the 50 J/cm^2^ group, serum LDH level increased from 49.7 to 84 IU/L on day 1 and decreased to 64.7 IU/L on day 7. In the 100 J/cm^2^ group, serum LDH level increased from 40.3 to 103 IU/L on day 1 and remained high at 117.7 IU/L on day 7.

Compared with the baseline values, the WBC count and CRP and LDH concentrations increased with irradiation dose. The WBC and CRP and LDH concentrations returned to the baseline levels in the 25 J/cm^2^ group but remained high or increased further in the 100 J group.

The Laserphyrin concentration before irradiation was 20–30 µg/ml in all dogs, except for a value of 62.3 µg/ml in one dog in the 50 J/cm^2^ group.

### Endoscopic Findings (Figures 2 and 3)

In the 25 J/cm^2^ group, a reddish color change was observed at the site of irradiation of the mucosa on day 1 in two dogs, and no change was detected in one dog. On day 7, ulceration was observed at the site of irradiation in the sites that showed redness on day 1, but only redness and erosion were recognized in the other dog.

**Figure 2:**
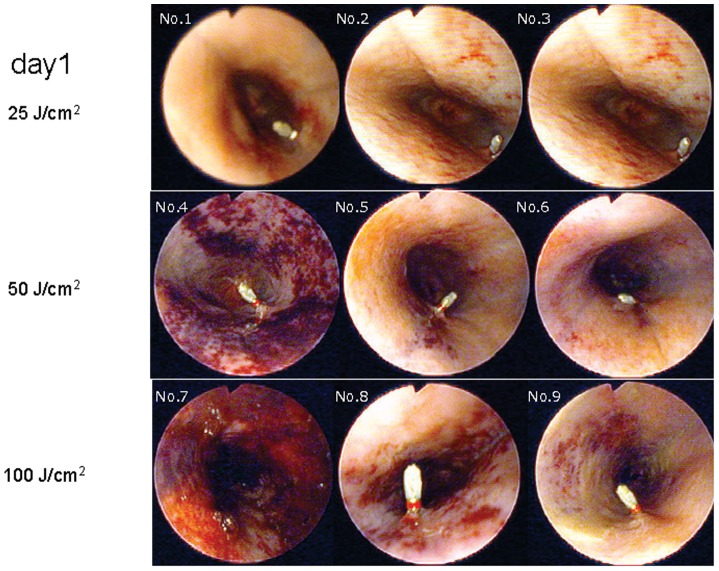
Endoscopic findings in the esophagus on postirradiation day 1. In all dogs except one in the 25 J/cm^2^ group, the mucosa at the site of irradiation (the area at 9 o’clock) showed reddish color changes on day 1 (A–C). Redness and ischemic color changes were observed at the site of irradiation in all dogs in the 50 J/cm^2^ and 100 J/cm^2^ groups (D–I).

**Figure 3:**
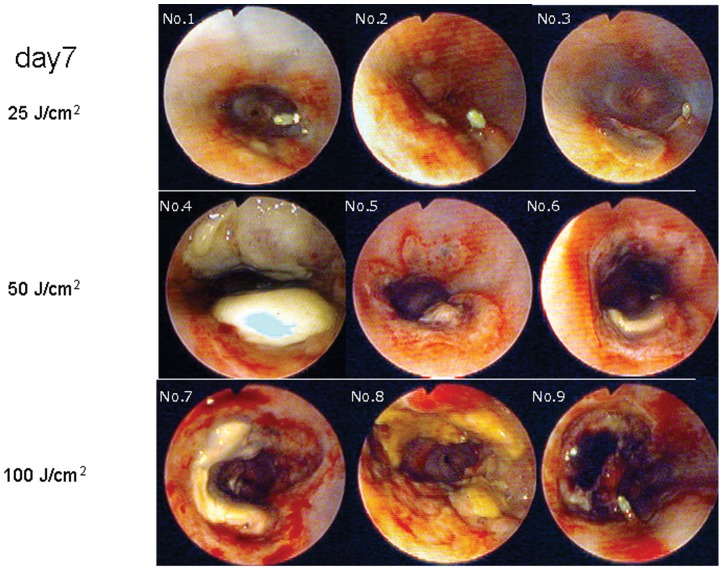
Endoscopic findings in the esophagus on postirradiation day 7. Redness and slight bleeding were observed at the site of irradiation in all dogs on day 7. Ulceration was observed in the 25 J/cm^2^ group (A–C) but was more extensive in both the 50 J/cm^2^ and 100 J/cm^2^ groups (D–I).

In the 50 J/cm^2^ group, redness and erosion were observed at the site of irradiation on day 1. Ulceration was observed on day 7 in all dogs, and purple color changes were observed at the ulcer base in two dogs.

In the 100 J/cm^2^ group, redness and erosion were observed at the site of irradiation in all dogs on day 1, and a blackish-purple color change was observed in one dog. On day 7, ulceration, redness, and erosion appeared in all dogs, and blackish-purple color changes occurred at the ulcer base in two dogs.

### Macroscopic Findings of the Autopsy (Figure 4 and Table 1)

In the 25 J/cm^2^ group, mucosal redness and erosion were observed in all dogs, and shallow ulceration was observed in two. No damage was observed at the outside of the esophageal wall in any case.

**Figure 4:**
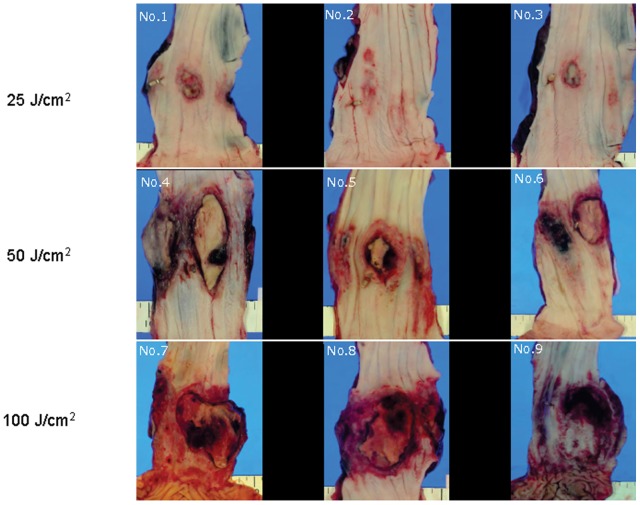
Macroscopic findings in the esophagus. Mucosal redness and ulceration were observed in all dogs in the 25 J/cm^2^ group (A–C). The areas of redness, erosion, and ulceration were more extensive in the 50 J/cm^2^ and 100 J/cm^2^ groups (D–I).

In the 50 J/cm^2^ group, ulceration with peripheral redness and erosion were observed in all dogs, and necrosis occurred in one. Adhesion of the esophageal adventitia to the aorta was recognized in all dogs.

In the 100 J/cm^2^ group, ulceration with peripheral redness and erosion were observed in all dogs, and necrosis appeared in two. Adhesion of the esophageal adventitia to the lungs was observed in all dogs, and adhesion to the aorta was detected in two dogs. The areas of tissue injury were 52±48 mm^2^, 498±431 mm^2^, and 831±692 mm^2^ after irradiation at 25 J/cm^2^, 50 J/cm^2^, and 100 J/cm^2^, respectively.

### Pathological Findings (Table 2)

In the 25 J/cm^2^ group, only erosion was observed at the site of irradiation in one dog, and ulceration was recognized in the other two dogs. Cellular infiltration was observed in the mucosa, submucosa, muscle layer, and adventitia in the 25 J/cm^2^ group and within and outside of the adventitia in the 50 J/cm^2^ and 100 J/cm^2^ groups. Fibrosis and hemorrhage were observed in the submucosa, muscle layer, and adventitia in the 25 J/cm^2^ group and within and outside of the adventitia in the 50 J/cm^2^ and 100 J/cm^2^ groups. Necrosis was observed in the submucosa and muscle layer in the 25 J/cm^2^ group and within and outside of the adventitia in the 50 J/cm^2^ and 100 J/cm^2^ groups. These changes tended to be more severe as the irradiation dose increased.

**Table 2:** Pathological findings in the injury areas.

Laser irradiation		25 J/cm2			50 J/cm2			100 J/cm2		
Animal number		1	2	3	4	5	6	7	8	9
Cellular infiltration	Mucosa	+	+	+	+	2+	+	–	–	+
	Submucosa	2+	2+	2+	2+	2+	3+	–	3+	2+
	Muscle layer	+	+	+	2+	2+	3+	2+	2+	2+
	Adventitia	2+	+	+	2+	2+	3+	3+	3+	2+
	Out of Adventitia	–	–	–	2+	+	2+	2+	2+	2+
Fibrosis	Mucosa	NE	NE	NE	NE	NE	NE	NE	NE	NE
	Submucosa	+	+	+	2+	2+	+	–	–	+
	Muscle layer	2+	2+	2+	3+	2+	2+	2+	2+	2+
	Adventitia	+	–	–	2+	2+	2+	2+	3+	2+
	Out of Adventitia	–	–	–	+	+	2+	2+	2+	2+
Hemorrhage	Mucosa	NE	NE	NE	NE	NE	NE	NE	NE	NE
	Submucosa	2+	±	+	2+	2+	2+	–	–	2+
	Muscle layer	+	±	+	2+	2+	2+	2+	+	2+
	Adventitia	+	±	±	2+	2+	2+	2+	2+	3+
	Out of Adventitia	–	–	–	2+	+	2+	2+	2+	3+
	Aorta	–	–	–	–	–	+	–	–	–
Necrosis	Mucosa	–	–	–	3+	–	–	–	–	–
	Submucosa	2+	+	+	3+	2+	3+	3+	3+	2+
	Muscle layer	2+	–	+	3+	2+	3+	3+	3+	3+
	Adventitia	NE	NE	NE	NE	NE	NE	NE	NE	NE
	Out of Adventitia	–	–	–	–	+	+	+	2+	2+
	Aorta	–	–	–	–	–	+	–	–	–

Notes) -: No abnormal changes, ±: Very slight, + : Slight, 2+: Moderate, 3+: Marked, NE: Not examined.

Cellular infiltration was observed in the mucosa, submucosa, muscle layer, and adventitia in the 25 J/cm^2^ group. Cellular infiltration was observed within and outside of the adventitia in the 50 J/cm^2^ and 100 J/cm^2^ groups. Fibrosis and hemorrhage were observed in the submucosa, muscle layer, and adventitia in the 25 J/cm^2^ group, and fibrosis and hemorrhage were observed within and outside of the adventitia in the 50 J/cm^2^ and 100 J/cm^2^ groups. Necrosis was observed in the submucosa and muscle layer in the 25 J/cm^2^ group and within and outside of the adventitia in the 50 J/cm^2^ and 100 J/cm^2^ groups. These changes tended to become more severe as the irradiation dose increased.

No infiltration of inflammatory cells, fibrosis, hemorrhage, or necrosis was observed in the extra-adventitial tissue in the 25 J/cm^2^ group. These changes were seen in the 50 J/cm^2^ and 100 J/cm^2^ groups, and they tended to be more severe as the laser dose increased. In one dog in the 50 J/cm^2^ group, hemorrhage from the aorta, which had adhered to the esophagus, was accompanied by necrosis. Inflammatory changes and necrosis of the extra-adventitial tissue were observed histologically in the 100 J/cm^2^ group.

## Discussion

The effects of Photofrin PDT, a first-generation PDT, depend on the selective retention of the photosensitizer in the neoplastic tissue. Laserphyrin PDT, a second-generation PDT, induces a phototoxic effect when the timing of photoactivation is associated with the peak plasma level of the photosensitizer. Therefore, in normal tissue irradiated by Laserphyrin photoactivation, phototoxicity would be expected even in normal tissue. The relationship between tissue damage and the dose of laser irradiation by Laserphyrin photoactivation remains unknown in the esophagus. To apply Laserphyrin PDT as a treatment option for esophageal cancer, the relationship between the laser irradiation dose and the damage to normal tissue and neoplastic tissue must be known.

In the present study, we evaluated, for the first time, the safety of Laserphyrin photoactivation in the normal esophageal wall. This *in vivo* study using a canine model showed that increasing the dose of Laserphyrin irradiation from 25 J/cm^2^ to 100 J/cm^2^ caused more severe inflammatory changes, as shown by the laboratory data, macroscopic damage, and histological necrosis. Macroscopically, the ulceration and ischemic changes around the ulcer became more severe as the dose of laser irradiation increased. Pathologically, these changes were confined to the mucosa after irradiation at 25 J/cm^2^, whereas they appeared as necrosis in the muscle layer after irradiation at 50 J/cm^2^, and the necrosis extended to the extra-adventitial tissue after irradiation at 100 J/cm^2^.

Adverse effects, such as appetite loss, nausea, increased inflammatory reaction, and an increase in LDH level, were confirmed, and these were related to the degree of tissue injury in the esophagus. Clinical symptoms were aggravated as the dose of laser irradiation increased. Considering these effects together, we conclude that an irradiation energy of 25 J/cm^2^ was within the safe range of Laserphyrin photoactivation in the canine normal esophagus. Tissue fragility differs between humans and dogs, and this dosage cannot be applied directly to humans. Previous data on the normal canine bronchi and a clinical study of human lung cancer suggest that similar effects should be expected in humans after double-irradiation doses that were used in dogs (unpublished data). Therefore, we recommend that human clinical trials in the esophagus should start with an irradiation dose of 50 J/cm^2^, which is similar to that used to treat lung cancer.

Laserphyrin PDT is expected to have a greater effect on the deep-tissue layers compared with Photofrin PDT [13], [14]. Laserphyrin PDT provides excellent clinical treatment responses in patients with early lung cancer [15]–[17]. Laserphyrin PDT might produce similar clinical effects when used to treat esophageal disease, especially for salvage treatment after CRT for esophageal cancer. If so, this would be advantageous for patients because of the lower risk of phototoxicity and shorter interval of light shielding.

This study has some limitations because we could not evaluate the actual tumoricidal effect on esophageal cancer. However, it is difficult to simulate exactly the human pathology and treatment in an animal model. Tissue thickness and fragility differ between humans and animal models. Establishing an esophageal cancer model is difficult in a large animal model. To evaluate the direct effects of Laserphyrin PDT on human esophageal cancer, we carefully run a well-controlled clinical trial. Another limitation was that we did not evaluate the effect by dose escalation of Laserphyrin. However, the recommended timing of the irradiation in humans has been established at a concentration of 20 µg/ml in the plasma. Therefore, we believe that there is no need to estimate another dose in clinical applications.

In conclusion, we have for the first time determined the safety range of 25 J/cm^2^ for irradiation with a diode laser in the canine esophagus when the plasma concentration was around 20 µg/ml. This information will be useful for planning clinical studies to examine the effects of Laserphyrin PDT on various esophageal diseases.

## Materials and Methods

### Animals

Male Beagle dogs weighing 12.0–14.7 kg and 13–14 months old were used in this study. The site of laser irradiation was determined as the area at 9 o’clock 5 cm oral to the esophagogastric junction, where it was possible to stably hold an endoscope and irradiate with a laser. The sites at 5 o’clock and 3 o’clock were marked in advance with a clip and tattooing as the landmarks for irradiation.

This preclinical research was done at the Shinn Nihon Biomedical Laboratories, Ltd. (Kagoshima, Japan) according to the Good Laboratory Practice Guideline. All research and experimental protocols were conducted according to the Regulation for the Care and Use of Laboratory Animals of Shinn Nihon Biomedical Laboratories and were approved by the Animal Care and Use Committee of Shinn Nihon Biomedical Laboratories, Ltd. (Approval ID: No. 704-006).

### Photosensitizer

Laserphyrin (Meiji Seika Pharma, Tokyo, Japan) is a water-soluble photosensitizer with a molecular weight of 799.69 and has a chlorine annulus. Its maximum absorption peak is at a wavelength of 407 nm, and there is a second peak at 664 nm. Laserphyrin has high tumor affinity and is excited by visible red light with a longer wavelength of 664 nm, enabling deeper penetration into the tissue [12], [13].

### Laser Unit and Endoscope

A diode laser (ZH-L5011HJP, Panasonic Health Care, Yokohama, Japan) emitting continuous-wave laser light at a wavelength of 664 nm was used as the laser source for the excitation of Laserphyrin. We used a straight-type fiber-optic tip for the irradiation. An Olympus GIF-XQ240 endoscope and a Panasonic PD laser device (ZH-L5011HJP) were used.

### Protocol of Laserphyrin Photoactivation in the Normal Esophagus

We fixed the dose of Laserphyrin because the profile of the systematic side effects is known and has been confirmed for lung cancer in a clinical study [14]. The approved dose of Laserphyrin for humans is 1 mg/kg, and the interval between Laserphyrin injection and laser exposure is 4–6 hours. In this condition, the blood concentration of Laserphyrin is about 20 µg/ml (unpublished data by Meiji Pharma, Tokyo).

In this animal model, Laserphyrin was administered intravenously into the cephalic vein or saphenous vein at a dose of 20 mg/kg. The time interval between photosensitizer injection and laser exposure was 60 min because the concentration of Laserphyrin in the blood during this interval was equal to that in humans (20 µg/dl) (data not shown).

To investigate the tissue damage in relation to the energy of the diode laser, dose escalation was set at 3 energy levels. The initial dose was set at an energy level of 25 J/cm^2^, and the second and the third were at 50 J/cm^2^ and 100 J/cm^2^, respectively. These three dosage groups comprised three dogs in each group.

**Figure 5:**
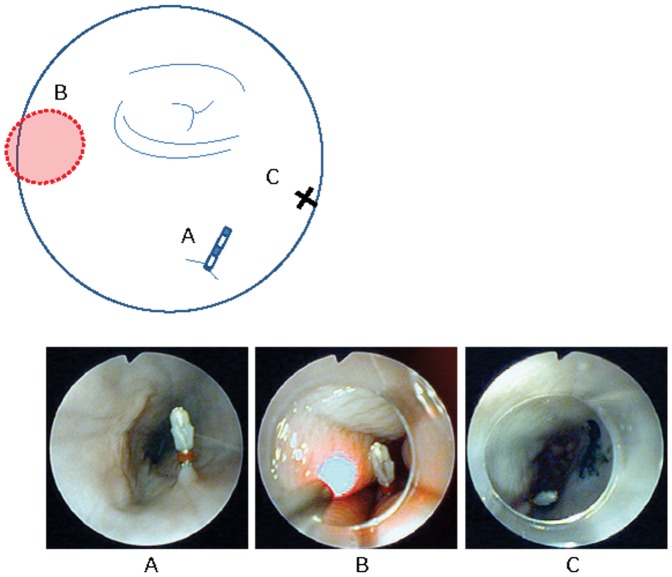
Standard operation procedure for laser irradiation. The site of laser irradiation was fixed at 9 o’clock (A) in all animals because this direction was easiest for holding an endoscope stably and irradiating with a laser. Before laser irradiation, the site at 5 o’clock (B) was marked in advance with a clip. After completion of the laser irradiation (C), the site at 3 o’clock (D) was tattooed to identify the irradiation site for follow-up and autopsy.

Laser irradiation was applied under general anesthesia using isoflurane. The target area was 1 cm^2^ in the normal esophageal mucosa in the 9 o’clock direction and 5 cm from the esophagogastric junction (Figure 5). To avoid missing the location of the irradiated area, the sites at 5 o’clock and 3 o’clock were marked in advance with a clip and tattooing by Indian ink, respectively, as the landmarks for irradiation. The dogs were fasted, and only water was given on the day of irradiation (day 0) and the next day (day 1), and a liquid meal was fed from day 2.

### Evaluation of the Effect of Laserphyrin Photoactivation in the Normal Esophagus

To evaluate the response to Laserphyrin photoactivation, changes on the surface of the whole esophagus including the irradiated area were examined endoscopically on days 0, 1, and 7. After the endoscopic examination on day 7, pentobarbital sodium was injected intravenously to provide anesthesia, and the dogs were exsanguinated for euthanasia. The internal organs and tissues were observed macroscopically, fixed in formalin, and subjected to hematoxylin and eosin staining for pathology evaluation. To measure the Laserphyrin concentration in the blood, blood was collected at the time of laser irradiation, which was 60 min after administration.

To evaluate the systemic effects of Laserphyrin, blood was collected from the external jugular vein before irradiation (day 0) and on postirradiation days 1 and 7, and the inflammatory reactions and hematological adverse effects were examined. To determine whether there was reduced oral food intake, the amount of leftover food from the previous day was recorded from day 2 to day 7 and thereafter. Body weight was measured before irradiation on day 0 and on day 7.
